# Feminizing Genitoplasty in Congenital Clitoromegaly: A Case Report

**DOI:** 10.7759/cureus.68680

**Published:** 2024-09-04

**Authors:** Preeti Gattani, Sakshi Heda, Aarzoo S Kedar, Rasika D Zade, Rajesh G Gattani

**Affiliations:** 1 Obstetrics and Gynaecology, Datta Meghe Medical College, Datta Meghe Institute of Higher Education and Research (Deemed to be University), Wardha, IND; 2 Obstetrics and Gynaecology, All India Institute of Medical Sciences, Rishikesh, IND; 3 General Surgery, Jawaharlal Nehru Medical College, Datta Meghe Institute of Higher Education and Research (Deemed to be University), Wardha, IND

**Keywords:** ambiguous genitalia, clitoromegaly, clitoroplasty, female pseudo-hermaphrodite, feminizing genitoplasty, macroclitoris

## Abstract

Congenital clitoromegaly, also known as macroclitoris, is a rare congenital disorder. It is a cause of poor self-esteem, anxiety, and gender self-perception. It negatively affects social, romantic, and emotional domains. We present a case of female pseudohermaphroditism with congenital clitoromegaly in a 23-year-old woman who attained late menarche at 22 years of age. Her karyotype was normal (46XX). She had clitoromegaly and a small vaginal opening with labial fusion and scrotalization. We performed feminizing genitoplasty, which included neurovascular sparing clitoroplasty, labioplasty, and vaginoplasty. Follow-up after two weeks revealed feminine genitalia. She is delighted as her ambiguity has been corrected with a good cosmetic effect and is satisfied with the aesthetics, sexual arousal, and self-esteem.

## Introduction

Clitoromegaly, also called macroclitoris, is an abnormally large clitoris. It is equivalent to the penis in males [1]. The clitoris has about 8,000 nerve endings responsible for producing pleasure. Clitoromegaly is a rare gynecologic condition [1,2]. It is a distressing situation for parents and relatives, especially if present at birth. The phenotypic appearance of ambiguous external genitalia raises suspicion of gender [2].

According to Brodie, clitoromegaly in adults is defined by a hood length exceeding 27.4 mm and a width of 8 mm [1,3]. A clitoral area of more than 35-45 mm^2^ is also considered to be clitoromegaly [4-6]. It can be congenital or acquired. The incidence is 1:10000 to 1:20000 for females [1]. The mean clitoral length at birth in Indians is 3.1 mm [7]. A clitoral length of more than 10 mm is considered clitoromegaly in a newborn girl [8]. Obvious clitoromegaly with ambiguous genitalia is easily identifiable. Borderline conditions go unnoticed [9]. Clitoromegaly can be due to various causes: hormonal, nonhormonal, idiopathic, or pseudo-clitoromegaly [10]. The diagnosis of the exact cause of clitoromegaly involves an exhaustive workup of history, examination, serum hormonal tests, imaging, enzymatic, and genetic studies [11]. Congenital adrenal hyperplasia (CAH) is also a rare cause of clitoromegaly. The incidence of CAH is one in 15,000-16,000 live births worldwide [12]. It is caused by congenital insufficiency of the enzyme 21 hydroxylase [13]. Only 11% of all patients with CAH develop clitoromegaly [12].

Irrespective of the cause of clitoromegaly, the condition has severe and detrimental effects on mental health [14,15]. Due to the presence of ambiguous genitalia, the parents and relatives become aware of the conflict between the assigned sex of the baby and the actual appearance of the genitalia. This has a great psychological and psychosocial impact. It is a cause of great mental agony for the family. There is an overall alteration of body self-image with short stature, increased weight, and hirsutism [15]. This leads to social withdrawal, especially in team sports and medical examinations involving nudity. They avoid romantic and sexual relationships, although there is no net effect on pleasurable sexual activity [14,15].

## Case presentation

A 22-year-old unmarried woman visited our outpatient department with a history of enlargement of the clitoris since birth, which was initially small and gradually attained the present size of 4.5 cm by 1 cm. She also complained of gradual narrowing of the vaginal orifice, which she noticed in the past two years. She has increased facial hair, which requires cosmetic removal every two to three days for the past four years. She had a delayed menarche at the age of 22. Her menstrual cycles have been normal since last year, with an average flow of five days. There was no history of drug abuse.

**Figure 1:**
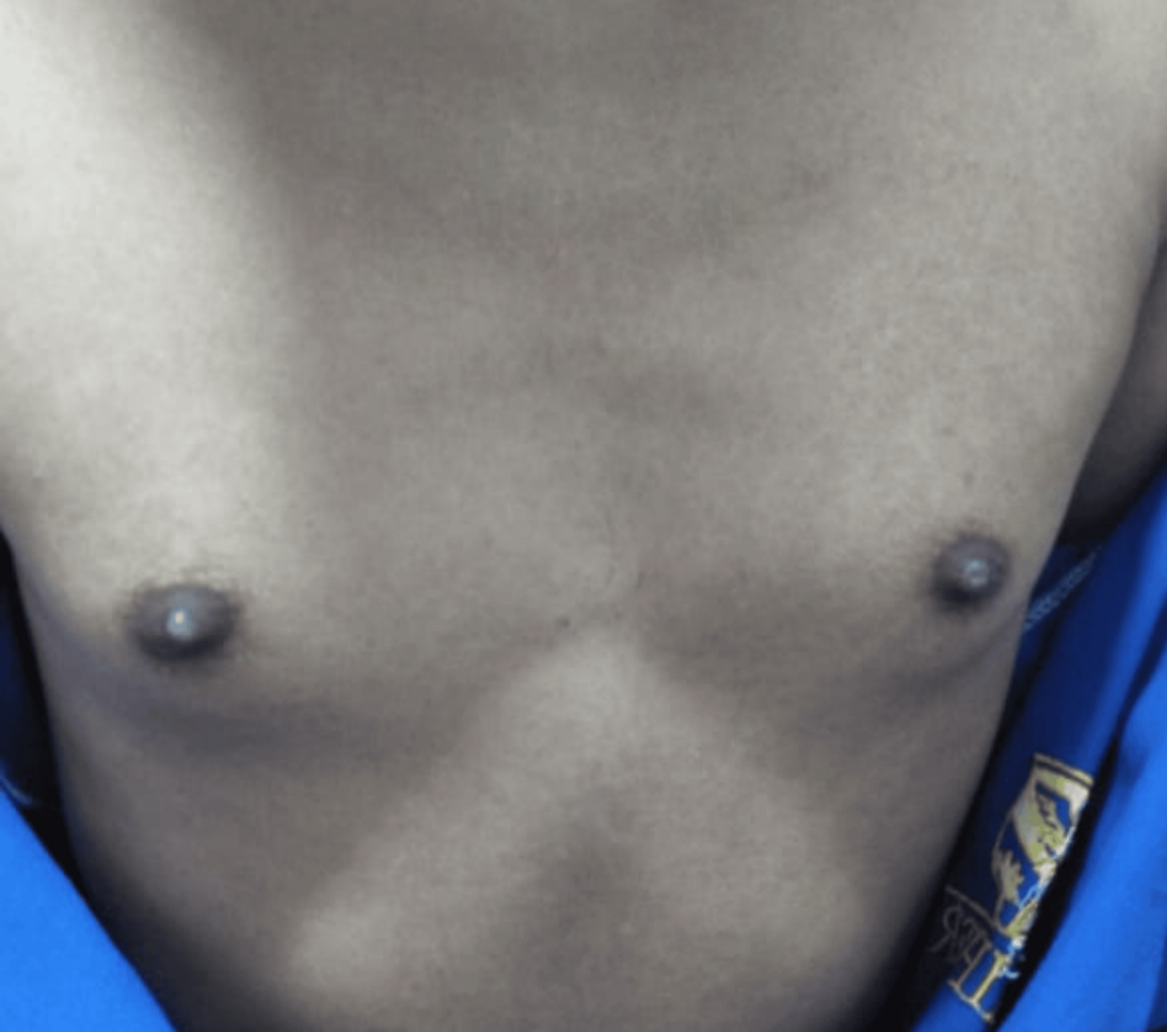
Breast development, Tanner stage 3.

There was no family history of clitoromegaly and no history of clitoral irritation secondary to masturbation or other sexual functions. The patient is thinly built. Her height was 137.16 cm and weight was 41 kg. Secondary sexual characters were phenotypic of males. Breast development, according to Tanner, was grade 3, as shown in Figure 1.

**Figure 2:**
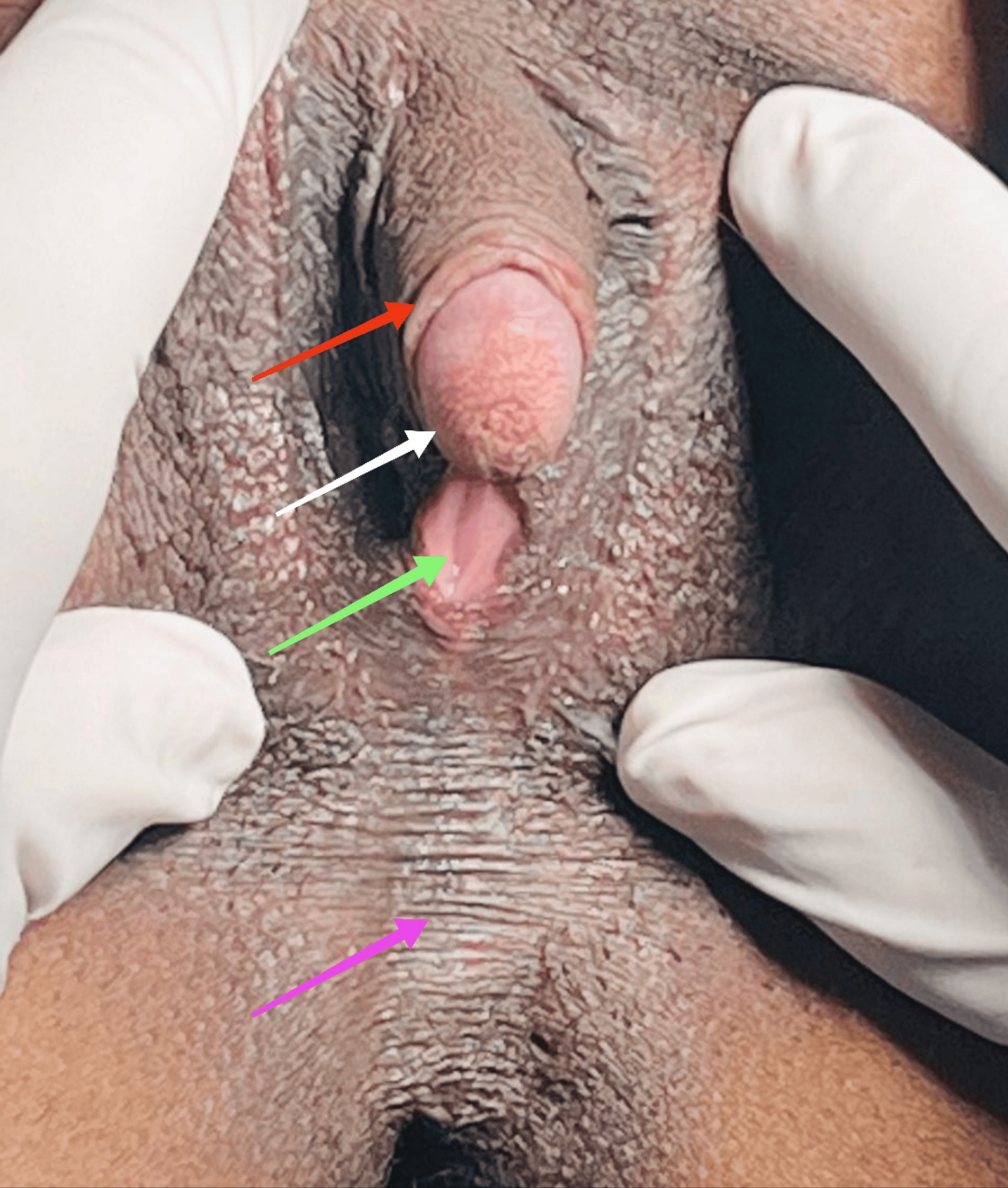
Clitoromegaly resembling a penis. The red arrow shows the prepuce; the white arrow indicates the glans penis; the green arrow shows occluded introitus due to scrotalization; and the purple arrow shows median raphe with scrotalization.

Pubic hair and axillary hair were normal. On local examination of the genitalia, the clitoris was enlarged, resembling a penis. The micropenis measured 6 cm x 2 cm x 1 cm after stretching and 4.5 cm x 2 cm x 1 cm without stretching in the non-arousal state. It had retractable prepucial skin and a hooded prepuce, as shown in Figure 2.

**Figure 3:**
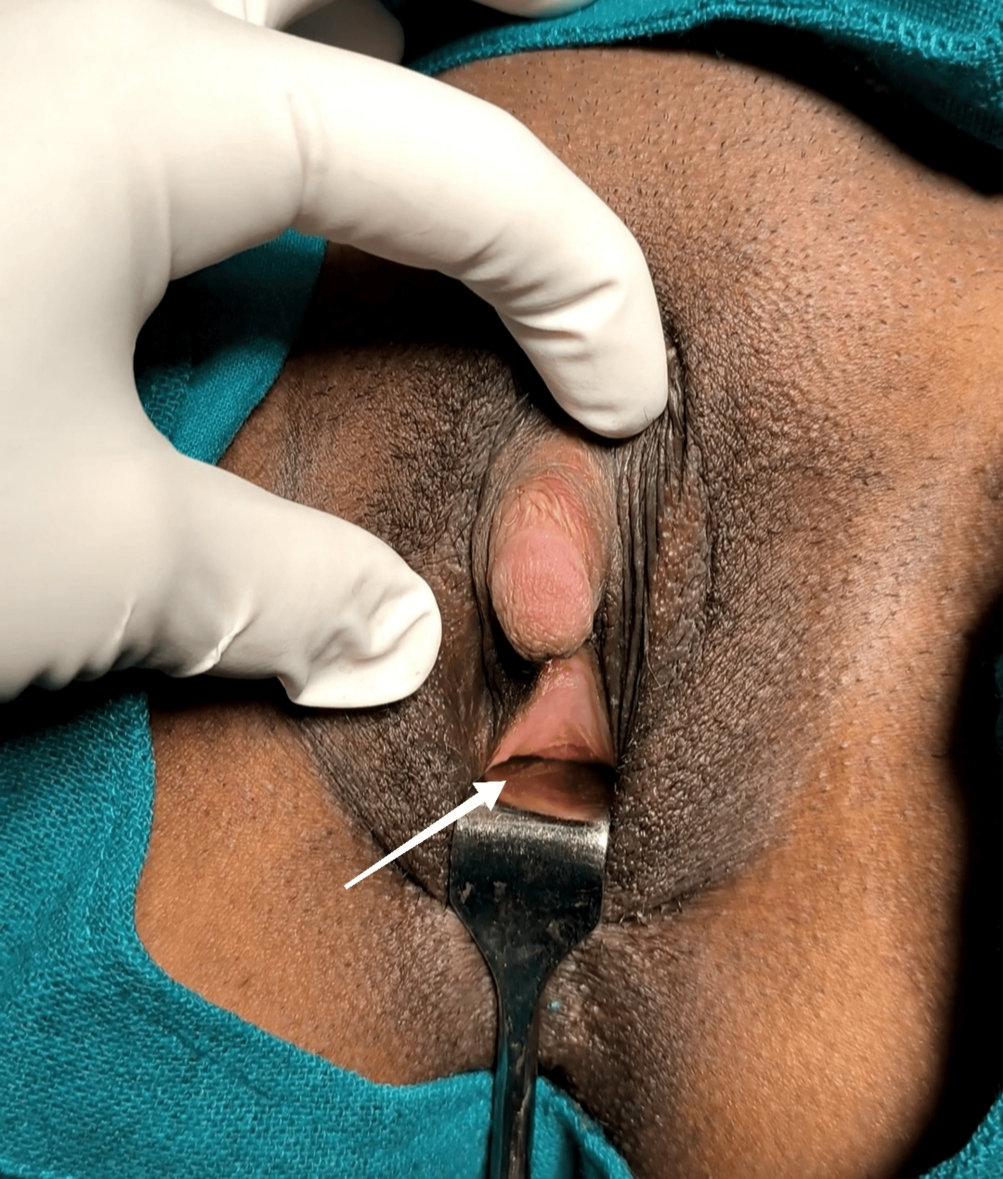
Narrow vaginal opening. The white arrow shows the small vaginal opening only seen with the help of speculum.

The labia majora were fused at the fourchette posteriorly, and there was scrotalization and rugosity of the skin of the secondary fused labia. The median raphe is ill-formed. The labia minora were redundant. The urethral opening was normally present. There were no hypospadias or epispadias. The vaginal opening was about 5 mm, which was reduced because of the formed scrotum covering the vagina, as shown in Figure 3.

The vaginal canal was ‘L’ shaped due to scrotalization and fusion of the labia majora. Table 1 presents the investigations carried out.

**Table 1:** Investigations. Hb: hemoglobin; RBC: red blood cell; WBC: white blood cell; DLC: differential leukocyte count; PT: prothrombin time; INR: international normalized ratio; ALT: alanine aminotransferase (also known as glutamate pyruvate transaminase (GPT)); AST: aspartate aminotransferase (also known as glutamic oxaloacetic transaminase (GOT)); LFT: liver function test; FBS: fasting blood sugar; PMBS: postprandial blood sugar; M: male; F: female; Ped: pediatric.

Investigation	Values	Reference values
Hb%	12.3 gm%	M: 13-15.5 gm%; F: 12-14.5 gm%
Total RBC count	6.08 million/cu.mm	M: 4.5-6 million/cu.mm; F: 4.5-6 million/cu.mm
Total WBC count	9,200/cu.mm	M and F: 4,000–11,000/cu.mm
DLC (polymorphs)	62%	M: 40-80%; F: 40-80%; Ped: 15-80%
DLC (lymphocytes)	30%	M: 10-20%; F: 10-20%; Ped: 15-20%
PT (INR and index)	15.3 sec	Control: 13.0 sec
ALT (GPT)	12.9 IU/L	M (adult): <50 IU/L; F (adult): <35 IU/L
AST (GOT)	15.4 IU/L	M (adult): <50 IU/L; F (adult): <35 IU/L
Alkaline phosphatase	56.8 IU/L	30-120 IU/L
Protein, serum	7.17 g/dL	6.6-8.3 g/dL
Bilirubin, total	0.43 mg/dL	0.3-1.2 mg/dL
Creatinine, serum	0.9 mg/dL	M: 0.72-1.18 mg/dL; F: 0.55-1.02 mg/dL
Potassium (K+) - serum	3.2 mEq/L	3.5-5.1 mEq/L
Sodium (Na+) - serum	137 mEq/L	136-146 mEq/L
Urea, serum	37.73 mg/dL	17-43 mg/dL
Pus cell	Occasional	NA
FBS	88 mg/dL	70-100 mg/dL
PMBS	138 mg/dL	<140 mg/dL
Serum cortisol (8 am)	74.33 ng/mL	54.94-287.5 ng/mL

**Figure 4:**
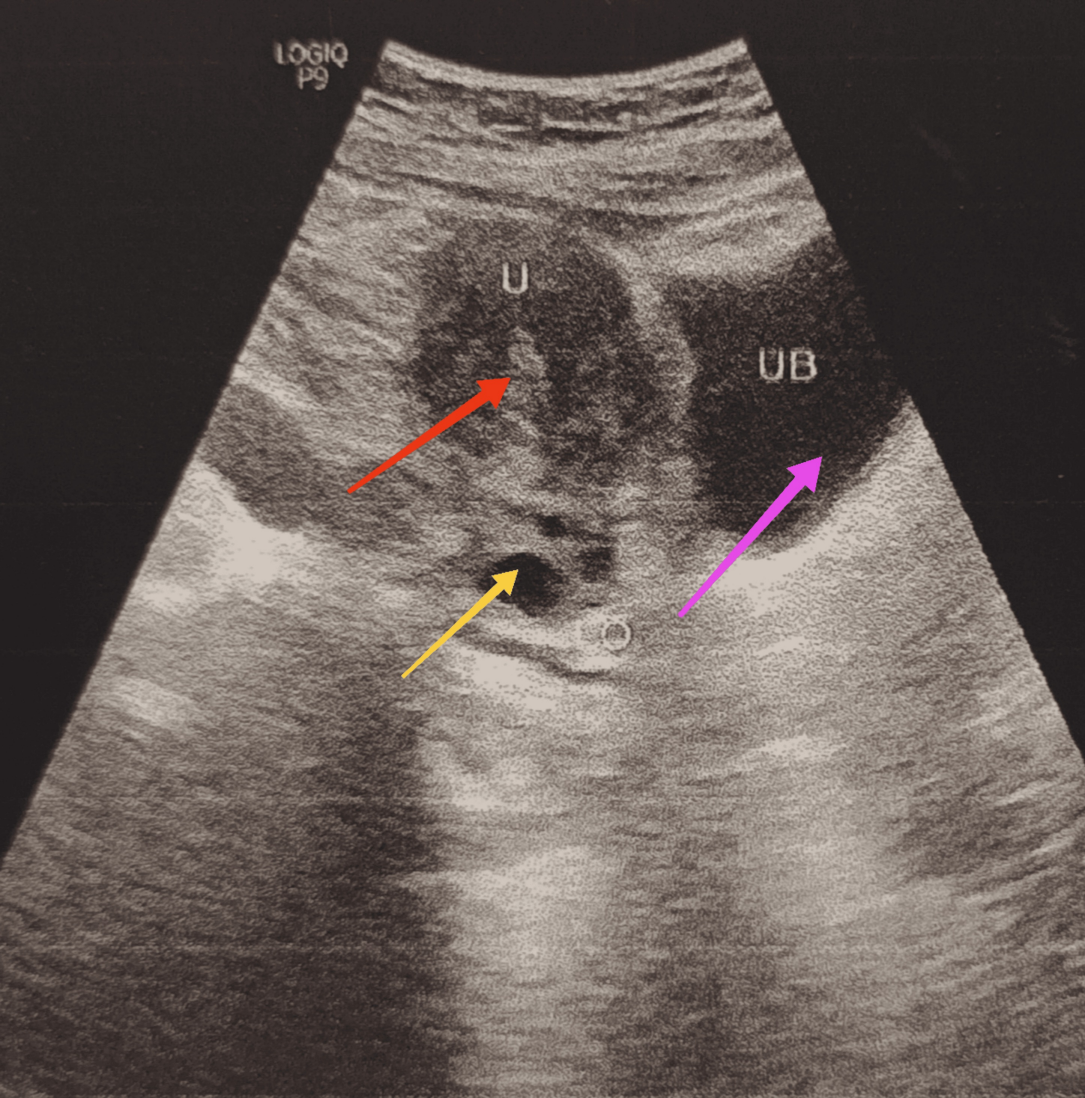
Sonography showing uterus and ovary. The red arrow shows the endometrium; the yellow arrow shows the left ovary; and the purple arrow shows the urinary bladder.

Her karyotype was 46XX. Her basic investigations were within normal range. Her sonography revealed normal adrenal size. The uterus was normal in size, measuring 5.8 cm by 2.3 cm by 3 cm, and both ovaries were normal, as shown in Figure 4.

The right ovary measured 3.1 cm by 1.4 cm, whereas the left measured 3 cm by 2 cm with a dominant follicle. The endometrial thickness was 6.3 mm. Magnetic resonance imaging revealed normal adrenals. The clitoral area was 507 mm^2^ in the sagittal plane (normal is 115±44 mm^2^). The distance of the clitoral body to the vaginal lumen was 9.4 mm, and the vagina was narrow. The length of the vagina was 6.9 cm There was a simple cortical cyst in the upper pole of the left kidney, measuring 1.4 cm by 1.3 cm. Her serum cortisol levels at 8:00 am were 74.33 ng/mL (normal 54.94-287.5). The endocrinologist prescribed tablet (Tab) Prednisolone 3.175 mg once daily.

**Figure 5:**
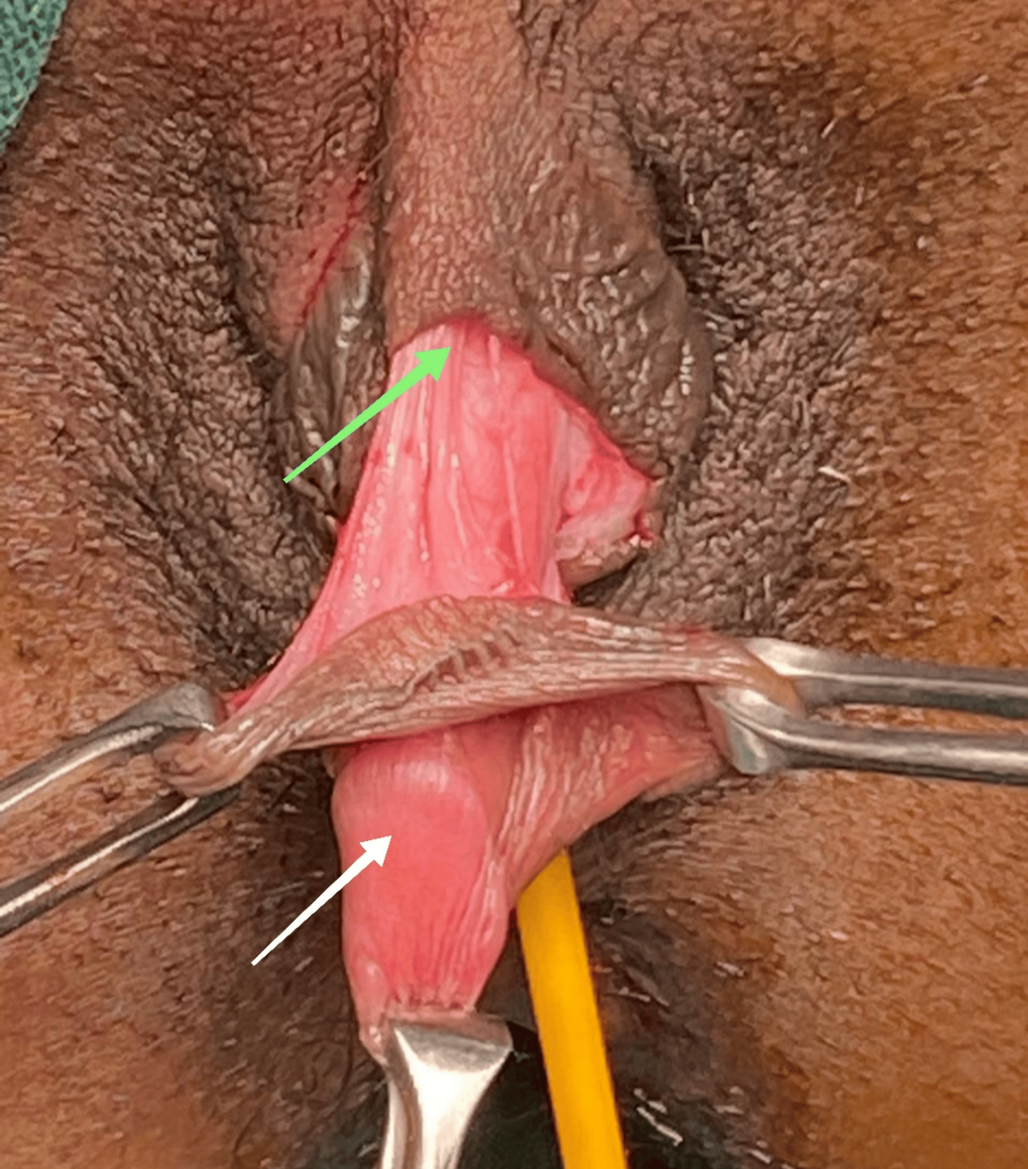
Incision for clitoroplasty. The green arrow shows the circumferential incision and the white arrow shows the glans penis.

A cystoscopy was done, which revealed a normal urethral opening at a normal site that was not incorporated in the micropenis. The urethral plate was normal, and changes in cystitis were present. A vaginal examination revealed a smaller uterus, and the cervix was felt high up. Clitoroplasty was done by Spencer and Allen’s procedure. A circumferential incision was taken from 9 to 3, following a clock pattern, leaving 5 mm of skin, as shown in Figure 5.

**Figure 6:**
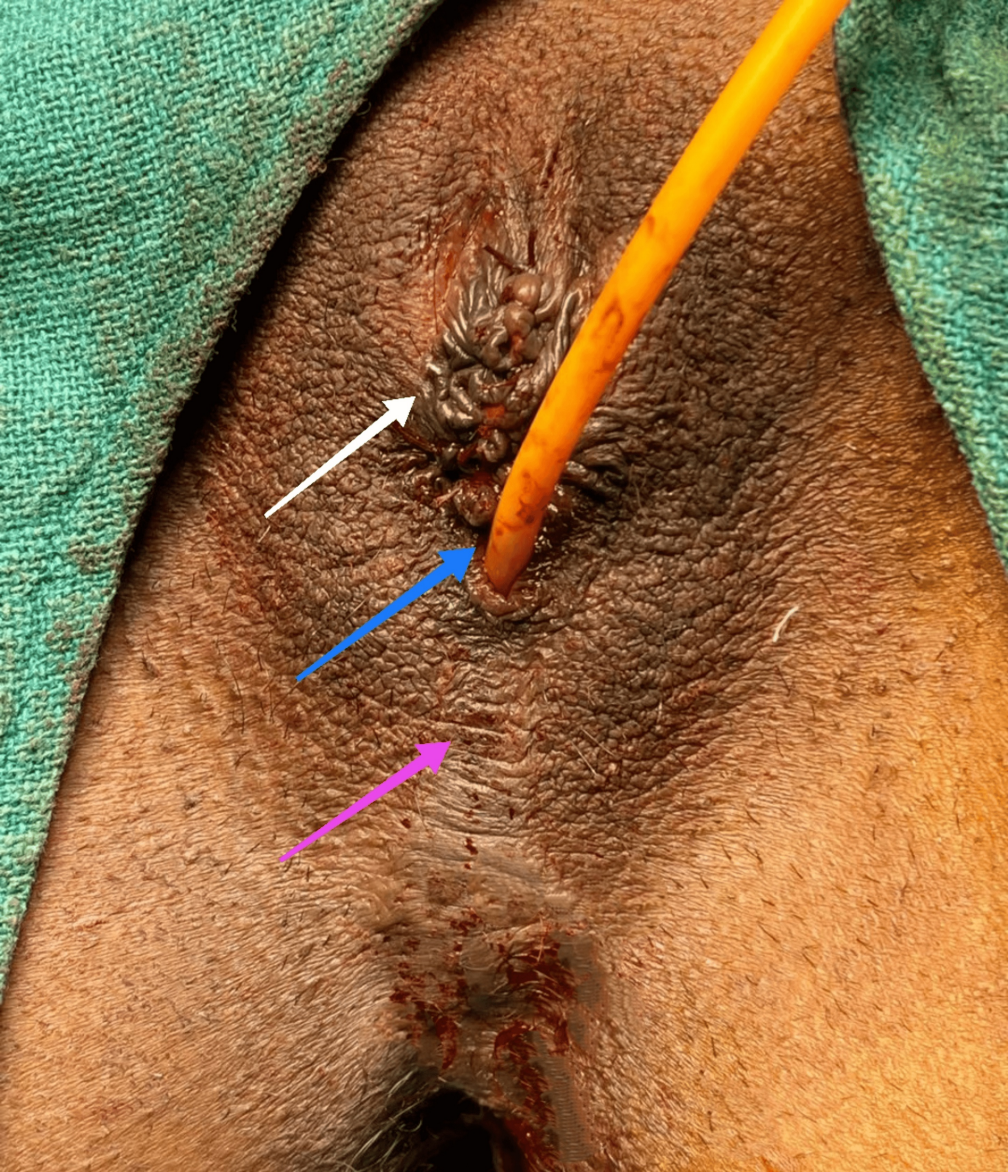
Completed clitoroplasty with catheter in urethra. The white arrow shows the neoclitoris; the blue arrow indicates the catheter in the urethra; and the purple arrow shows the scrotalized skin completely covering the vagina.

After the dissection of the cavernous and clitoris planes, the neurovascular bundle was isolated. The cavernous body was isolated around 4 cm and was excised to preserve part of its base and neurovascular bundle. The distal and proximal planes were sutured using Vicryl 3-0. Buck's fascia was fixed. The skin was then closed after achieving proper hemostasis, as shown in Figure 6.

**Figure 7:**
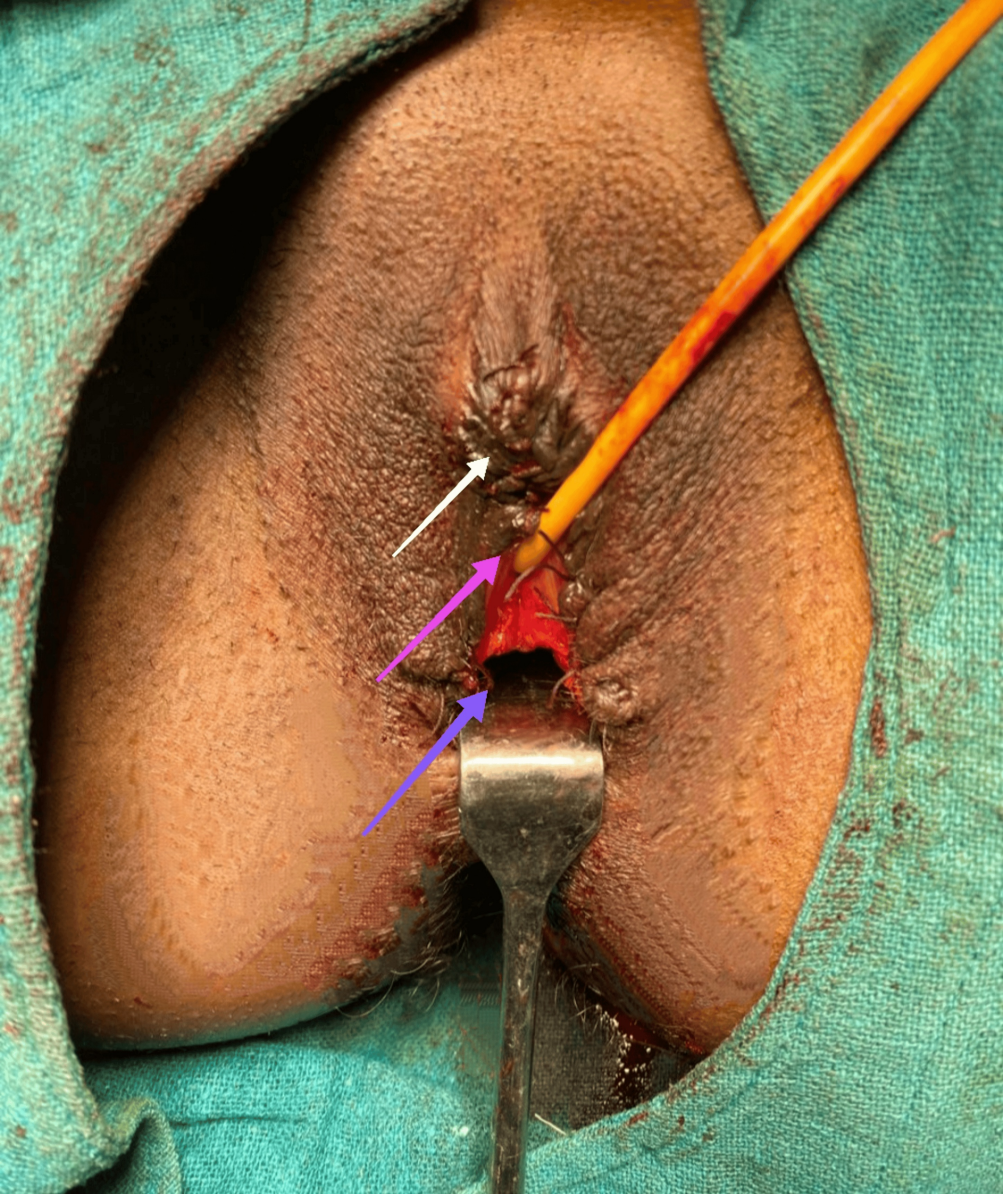
Completed labioplasty and vaginoplasty. The white arrow shows neoclitoris; the purple arrow shows the normal urethral orifice with the indwelling catheter; and the blue arrow shows the neovaginal opening after the completion of labioplasty and vaginoplasty.

Vaginoplasty was then done by removing the fused, scrotalized skin, as shown in Figure 7.

**Figure 8:**
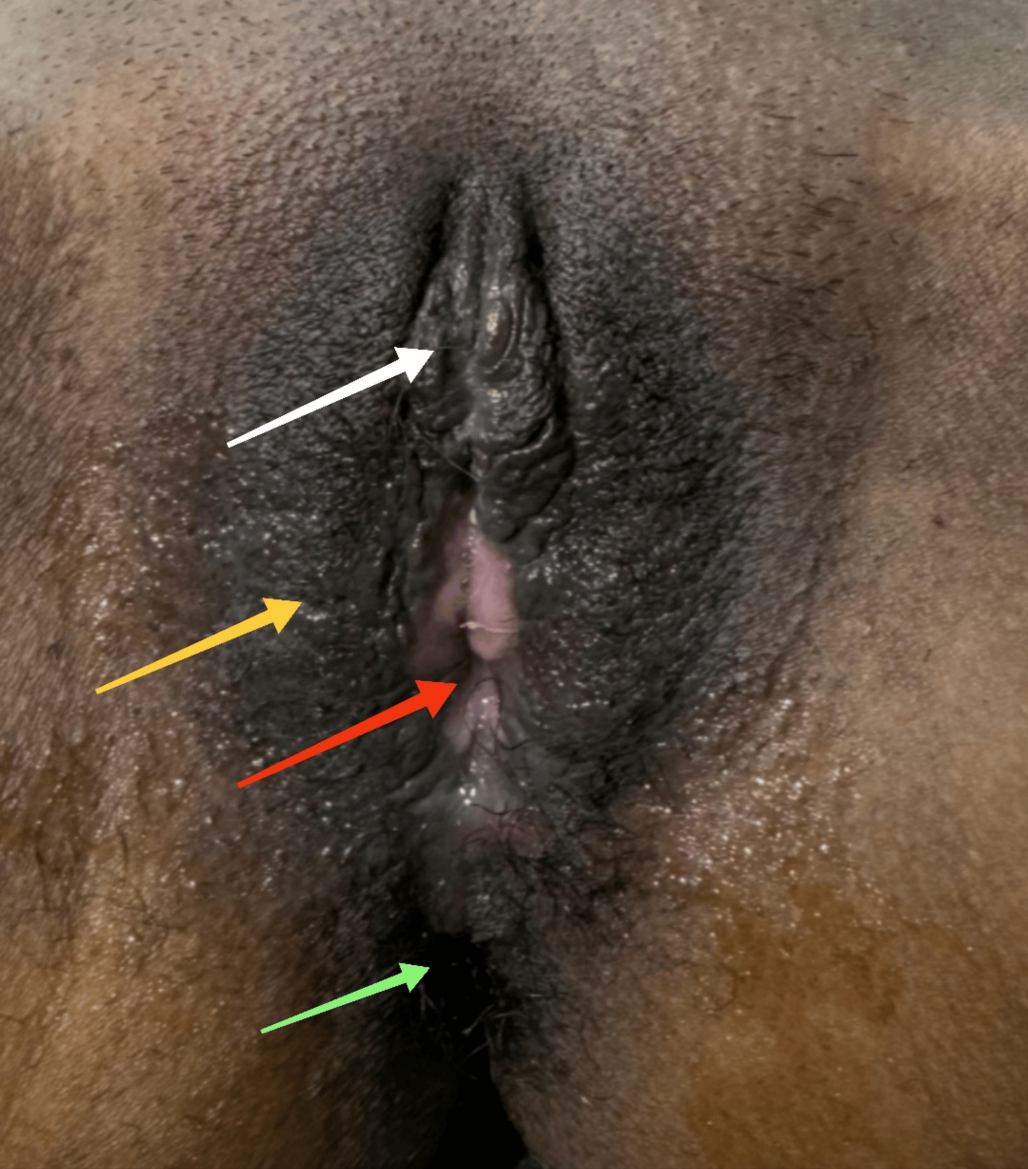
The feminine genitalia with good cosmetic effect at follow-up. The white arrow shows normal neoclitoris; the yellow arrow shows the neo-labia majora; the red arrow shows the introitus after vaginoplasty; and the green arrow shows the anal sphincter.

The vaginal anatomy was restored after stitching of the vaginal mucosa to the edge of the refreshed labia majora skin. She was discharged after five days. There were no early or late post-operative complications at follow-up. After one month, the sensation was normal, and the patient was delighted, happy, and extremely satisfied with the aesthetical and functional results, as shown in Figure 8.

## Discussion

The clitoris is a highly complex organ and is essential for sexual arousal [9]. Clitoromegaly is defined as abnormal enlargement of the clitoris. According to Brodie, in an adult, a hood length of more than 27.4 mm and a width of 8 mm are necessary to call it clitoromegaly [1,3]. Alternately, a clitoral area of more than 35-45 mm is considered as clitoromegaly [4-6]. It can be congenital when it has been present since birth, as in our case, or it can be acquired later in life.

The causes of clitoromegaly may be hormonal conditions, nonhormonal, pseudoclitoromegaly, or idiopathic [10]. The hormonal conditions can be due to endocrinopathies, masculinizing tumors, exposure to androgens, and various syndromes [10]. Neurofibromatosis, epidermoid cysts, abscess vulvitis, leiomyoma, nevus, and many syndromes are the nonhormonal causes of clitoromegaly [10]. Pseudoclitoromegaly can also be caused by masturbation [9]. The hormonal cause of virilization of female fetuses is also due to maternal exposure to androgens or androgenic drugs. Labioscrotal fusion and clitoromegaly occur if exposure to androgens occurs between eight and 14 weeks of pregnancy [9].

Pseudohermaphroditism is a cause of low self-esteem, poor gender self-perception, and poor self-perception. It has negative effects on nine of 10 life domains. Women are less likely to wear tight clothes, change clothes in public locker rooms, and play group sports [14]. Also, they have a net negative effect on plans for romantic and sexual relationships. All have an adverse effect, causing mental stress and disharmony. Studies show that there are no net positive effects of clitoromegaly. It has net negative effects on seven of 10 activities (p≤0.03) and no net effect (neutral) [14]. Clitoromegaly has net negative effects on nine of 10 life domains (p<0.001) and is neutral on job self-perception (p=0.25). Clitoromegaly has a negative psychological outcome and adds to the burden of living [14].

Our patient was diagnosed with congenital clitoromegaly and underwent a feminizing genitoplasty, which included a neurovascular sparing clitoroplasty, vaginoplasty, and valvuloplasty for an enhanced cosmetic look preserving her sexual arousal function and sensation. She also regained her self-esteem and has significantly improved her mental health. She was subsequently followed up after one month and three months. She is satisfied and has regained her self-esteem.

## Conclusions

Clitoromegaly is a rare condition that may be present at birth or may be acquired. It is associated with other medical diseases that require a scrutinized evaluation. Psychosocial consequences of atypical genital development are related to poor self-esteem, anxiety, and gender self-perception. Additionally, they are looked down on by society due to conflicting gender identification by family members. Pseudohermaphroditism also has negative effects on life domains. In such cases, it is advised to approach clinicians early so that any associated diseases can be diagnosed and treated and not shy away from such ambiguous genitalia.

Feminine genitoplasty is recommended to give a cosmetic look to the genitals. It is also imperative to maintain the sensitivity of the clitoris so that it allows sexual activity with pleasure, thus increasing her self-esteem and confidence, which helps in mental health management. It is also important to give a message about available corrective cosmetic surgeries.
